# Keystone bacteria dynamics in chronic obstructive pulmonary disease (COPD): Towards differential diagnosis and probiotic candidates

**DOI:** 10.1016/j.heliyon.2025.e42719

**Published:** 2025-02-14

**Authors:** Azadeh KavianFar, Hamidreza Taherkhani, Hossein Lanjanian, Sargol Aminnezhad, Ali Ahmadi, Sadegh Azimzadeh, Ali Masoudi-Nejad

**Affiliations:** a Laboratory of Systems Biology and Bioinformatics (LBB), Department of Bioinformatics, Kish International Campus, University of Tehran, Kish Island, Iran; b Software engineering department, engineering faculty, Istanbul Topkapi University, Istanbul, Turkey; c Laboratory of Systems Biology and Bioinformatics (LBB), Institute of Biochemistry and Biophysics, University of Tehran, Tehran, Iran; d Molecular Biology Research Center, Systems Biology and Poisonings Institute, Tehran, Iran; e Chemical Injuries Research Center, Systems Biology and Poisonings Institute, Tehran, Iran

**Keywords:** *COPD*, *Respiratory microbiome*, *Co-occurrence network*, *Keystone bacteria*, *Differential correlation*, *Pulmonology*

## Abstract

Preventing exacerbations in Chronic Obstructive Pulmonary Disease (COPD) is crucial due to the high mortality rate and the associated costs of hospitalization for patients during exacerbations. Despite the proven influence of the lung microbiome on disease control, the dynamics of bacterial communication in different stages of COPD remain unknown. This study aimed to propose a group of candidate bacteria for the differential diagnosis of different states of COPD based on the relative abundance correlation of bacteria in lung sputum samples. We compared microbiome data collected from 101 COPD patients in stable and exacerbation states, as well as 124 healthy controls from two separate general cohorts, to determine the major microbiome and keystone genera. To validate our findings, we utilized two additional distinct public datasets, each comprising 81 healthy subjects and 87 COPD patients in stable condition, exacerbation, and post-treatment phases. During COPD exacerbation, *Porphyromonas*, *Clostridium*, *Moryella*, and *Megasphaera* were identified as phenotype-specific keystone genera, while *Prevotella*, *Streptococcus*, *Haemophilus*, and *Veillonella* were consistently present across all datasets as core microbiome members. Changes in keystone genera during different COPD stages indicate rewiring of bacterial interactions, with increased keystone bacteria and network connectivity observed during dysbiosis and more severe COPD. *Bifidobacterium* showed probiotic potential, positively correlating with *Lactobacillus* during exacerbation, while *Neisseria* and *Haemophilus* increased in abundance, and negatively correlated with key probiotic bacteria. These findings indicate promising potential for the simultaneous use of *Bifidobacterium* along with *Lactobacillus* as a therapeutic candidate to prevent COPD exacerbations in lung health, underscoring the need for further research in future clinical studies.

## Highlights

• Lung microbiome plays a crucial role in the development of COPD.
• Changes in keystone genera across different COPD stages reflect a rewiring of bacterial interactions, with heightened keystone bacteria.
• As The severity of COPD increases, dysbiosis, the number of nodes, edges, and connectivity in the network also increase.
• *Lactobacillus* and *Bifidobacterium* are suggested as potential candidates for treating or preventing COPD exacerbations in the lungs in future clinical studies.

## Graphical abstract

**Figure:**
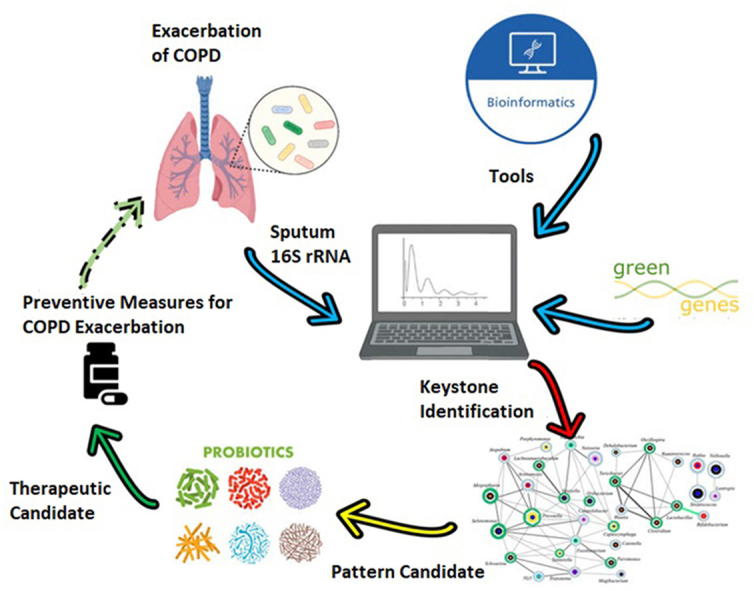

## 1 Introduction

Chronic Obstructive Pulmonary Disease (COPD) was recognized as the third leading cause of death by the World Health Organization (WHO) in 2019 [1]. COPD patients are prone to exacerbation (Acute Exacerbation COPD: AECOPD) mainly caused by bacterial or viral infections [2]. Some patients hospitalized for AECOPD for the first time die within a year of discharge [3]. These cases highlight the importance of further research on COPD to prevent or treat exacerbations.

The microbiome refers to the entire habitat, including the microorganisms (bacteria, archaea, lower and higher eukaryotes, and viruses) and their genomes [4]. Various factors can influence the composition and diversity of the microbiome, including sex, age, diet, geography, lifestyle, environmental exposures, and the use of antibiotics [5,6]. Understanding the importance of the relationship between microbes and the human body was the reason to conduct further research to unravel the role of the microbiome in controlling health and disease [7,8]. Changes in airway microbiome composition are linked to chronic lung diseases [9]. Recent studies showed that microbial diversity is a key feature of a “healthy” microbiome and the presence or absence of specific microbial taxa plays a critical role in disease [10]. The core microbiome is composed of traits that are present in the vast majority of the human microbiome [11]. The core microbiome identifies species functionally associated with diseases, which can be used to prevent or treat them [12]. The keystone bacteria identified in the respiratory microbiome of cystic fibrosis (CF) disease were anaerobic organisms [13]. By applying network topological evaluation criteria, the keystone species in co-occurrence networks can be predicted [14]. The keystone hypothesis proposes that some low-abundance microbes can have significant effects on the structure of the microbial community, changing a normal microbiome into a dysbiotic state [8].

A co-occurrence network of microbial communities may assist researchers in identifying the population subsets within a microbiome that interact in either a positive or negative manner [12,15]. The co-occurrence network is based on the mutual ecological correlation among microbial populations, which can be derived from taxonomic composition [16]. Co-occurrence networks can be used to assist disease diagnosis and prognosis [17]. Using microbial co-occurrence networks, we can learn more about the potential functional roles of a diverse range of key microbial taxa, not just in microbial communities, but also in host traits and physiological parameters [18].

16S ribosomal RNA (rRNA) sequences of prokaryotic organisms are reliable markers for taxonomic classification and phylogenetic analysis [19]. The sequence of the 16S rRNA gene comprises nine hyper-variable sections with varying degrees of conservation (V1-V9) [20,21]. High-throughput 16S rRNA gene sequencing showed that both the airways of healthy persons and patients have diverse, complex bacterial communities with overlapping compositions [10,22,23].

Considering that bacterial dysbiosis plays a role in disease exacerbation, and that COPD exacerbation is a factor affecting mortality, our primary objective in this study was to identify microbial candidates for preventive and therapeutic management of COPD exacerbations. To achieve this goal, we compared the composition of the lung microbiome and a network of bacterial genera in stable and exacerbated COPD states using 16S rRNA metagenomics sequencing data from lung sputum. Keystone genera in each state were identified based on network topology and biological criteria. The field of probiotic applications is rapidly evolving, extending beyond their initial use in gastrointestinal disorders [24,25]. Recently, probiotics [26,27], paraprobiotics [28], and lysates [29] showed promising results in various fields of medicine. Therefore, analyzing the probiotic potential in COPD patients by examining the differential correlations between bacterial pairs in the co-occurrence network across different COPD states, may help identify bacteria effective for treatment or prevention.

## 2 Materials and methods

**Fig. 1:**
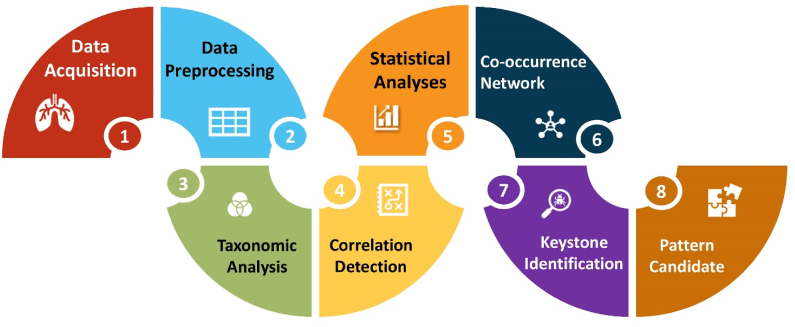
Graphical flowchart of the work process.

Fig. 1 displays a graphical representation of the work process, illustrating its various stages clearly. Subsequent sections will offer detailed explanations regarding each step outlined in the flowchart.

### 2.1 Data acquisition

Datasets, including sputum samples sequenced from the lung targeting the V4 region, were selected for COPD and healthy controls. We used two external datasets for healthy individuals and COPD patients for validation. The procedure for selecting and filtering datasets is shown in Fig. S1. In the healthy control dataset for validation, there were 304 samples of MZ twins, DZ twins, and their families. We chose one participant per family as the control group. These individuals had not taken antibiotics within the past three months and had no history of lung disease [30].

### 2.2 Data preprocessing

Microbiome data were analyzed using QIIME 2 (Version 2020.6, QIIME 2 Development Team, Northern Arizona University, Flagstaff, AZ, USA) [31]. For demultiplexing, dataset-specific sequence information, either single-end or paired-end, was created. After demultiplexing, denoising of the samples was performed. Sequence processing and error correction were performed using DADA2 (Callahan Lab, Stanford University, Stanford, CA, USA) [32]. The cleaned sequencing data were filtered, and samples with fewer than 5000 reads were excluded.

### 2.3 Taxonomic analysis

Operational Taxonomic Unit (OTU) clustering, assigning taxa, and analyzing feature tables are all performed as part of the taxonomic analysis in QIIME 2. Sequences with a similarity greater than 97 % were clustered into the same OTU using the de novo and closed-reference clustering approaches [33]. Taxonomic classification was performed using the Greengenes database with 99 % OTUs classifier (gg-13-8-99-nb-classifier.qza) via the QIIME 2 Plugin. Finally, diversity indices, such as alpha diversity (Shannon index and Faith_PD, shown in Figs. S3 and S4) [[34], [35], [36], [37], [38]], beta diversity (weighted UniFrac) [39,40], and Principal Coordinate Analysis (PCoA) [41,42], were performed using QIIME 2. The genus-level taxonomic data were selected for downstream analysis [43]. To avoid zero inflation, OTUs with zero frequency in more than 70 % of the samples were excluded. The relative abundance of all genera in each sample was calculated. Genera with a minimum frequency of 0.001 in more than 70 % of the samples were selected as core OTUs.

### 2.4 Correlation detection and statistical analysis

Spearman pairwise correlation coefficients were used between genera in each dataset separately using R (Version 4.2.1, R Foundation for Statistical Computing, Vienna, Austria). All pairs of correlations exhibiting a consistent trend during the disease exacerbation process were selected (Table 3). Subsequently, their significance was assessed to control for effects and to determine whether the correlation between two bacteria differed between states. The ANOVA model was employed for this analysis [44,45].

**Table 1:** Dataset and demographic properties of the samples. The data were downloaded from the EBI database and organized based on the type of biological sample (sputum sample) and the variable region within the 16S rRNA gene.

Access number	Disease	Final samples	Number of patients	Age Avg	Platform	Country
PRJNA491861	Healthy	122	124	61	Paired-end	Britain
PRJEB9607^a^	Healthy	101	81	58	Single-end	Korea
PRJNA377739	COPD (Stable& One Exacerbation& Two or more Exacerbation)	117 (39&38&40)	101	67	Paired-end	Britain
PRJNA299077^a^	COPD (Stable-Exacerbation-Post Therapy)	94 (21&36&37)	87	61	Single-end	Britain

a Independent External validation dataset.

**Table 2:** Taxonomic abundance and the core microbiome. The results reported in the healthy state and different states of the disease were common in both the main and validation datasets. The high relative abundance column displays genera with a frequency greater than 0.001. The low relative abundance column shows genera with a frequency lower than 0.001. The core microbiome includes genera whose relative abundance is greater than 0.001 in more than 70 % of the samples.

High relative abundance (above 0.001)			Low relative abundance (below 0.001)	Core microbiome
**Healthy**	***Streptococcus*** ***Prevotella*** ***Veillonella*** ***Rothia*** ***Neisseria*** ***Porphyromonas*** ***Leptotrichia*** ***Fusobacterium*** ***Haemophilus***	***Selenomonas*** ***Treponema*** ***Aggregatibacter*** ***Oribacterium*** ***Atopobium*** ***Capnocytophaga*** ***Campylobacter*** ***Actinomyces***	*Peptococcus* *Butyrivibrio* *Paludibacter* *Mycoplasma* *Cardiobacterium* *Dialister* *Kingella*	***Actinomyces*** ***Porphyromonas*** ***Prevotella*** ***Streptococcus*** ***Fusobacterium*** ***Leptotrichia*** ***Veillonella*** ***Campylobacter*** ***Neisseria*** ***Haemophilus*** ***Rothia***
**Stable COPD**	***Selenomonas*** ***Corynebacterium*** ***Campylobacter*** ***Moryella*** ***Aggregatibacter*** ***Oribacterium*** ***Moraxella*** ***Capnocytophaga*** ***Lactobacillus*** ***Leptotrichia***	***Porphyromonas*** ***Fusobacterium*** ***Actinomyces*** ***Veillonella*** ***Prevotella*** ***Neisseria*** ***Rothia*** ***Haemophilus*** ***Streptococcus***	*Tannerella* *Mogibacterium*	***Actinomyces*** ***Rothia*** ***Prevotella*** ***Streptococcus*** ***Veillonella*** ***Fusobacterium*** ***Neisseria*** ***Haemophilus***
**Exacerbation COPD**	***Selenomonas*** ***Corynebacterium*** ***Lactobacillus*** ***Capnocytophaga*** ***Porphyromonas*** ***Fusobacterium*** ***Actinomyces***	***Veillonella*** ***Prevotella*** ***Rothia*** ***Neisseria*** ***Moraxella*** ***Haemophilus*** ***Streptococcus***	*Mogibacterium* *Tannerella* *Lachnoanaerobaculum* *Parvimonas*	***Actinomyces*** ***Rothia*** ***Prevotella*** ***Streptococcus*** ***Veillonella*** ***Neisseria*** ***Haemophilus***

**Table 3:**
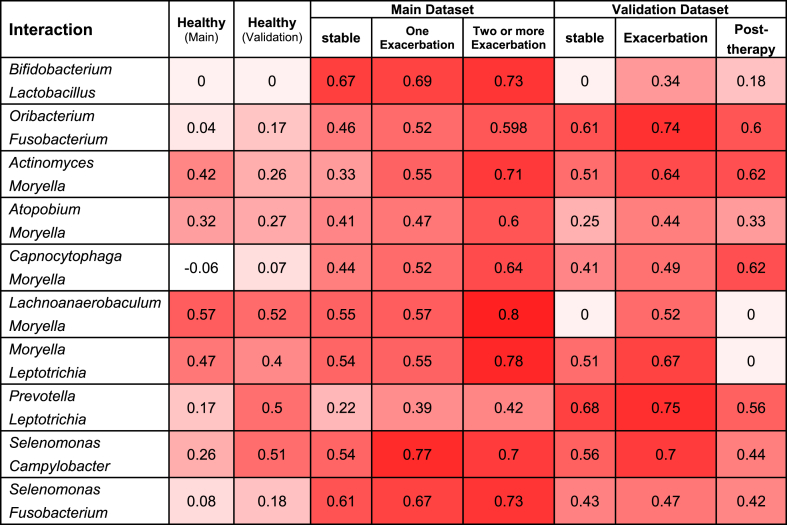
Differential correlations between healthy and disease state in the main and validation datasets.

### 2.5 Co-occurrence network

To construct the co-occurrence network, we filtered pairwise correlation coefficients (>|0.6|, p-value <0.01). Cytoscape (Version 3.9.0, Cytoscape Consortium, San Diego, CA, USA) was used for visualization. Network analysis was conducted to assess the differences in the number of nodes, number of edges, average clustering coefficient, and network connectivity between each state and the subsequent state.

### 2.6 Keystone identification

Keystone genera were determined using both topological and biological criteria. Here, we calculated five topological network properties for each node: Degree (the number of edges for each node), Clustering Coefficient (the likelihood of a node clustering with other nodes), Closeness Centrality (the average distance between a node and other nodes, determining how central it is in the network), Betweenness Centrality (the number of shortest paths that pass through the node), and Topological Coefficient (a measure of how many other nodes share neighbors with a specific node). For the biological index, we focused on anaerobic bacteria.

### 2.7 Pattern candidate

We investigated the relationship between significant differential correlations among pairs of bacteria during the disease exacerbation process (as shown in Table 3) and their interactions with other bacteria at each disease stage. Additionally, we examined their potential pathogenic effects throughout the disease progression.

### 2.8 Risk of bias

In conducting meta-analyses and systematic reviews, assessing the risk of bias (ROB) is essential, particularly in microbiome research, where studies often have low sample sizes and limited statistical power. The high variability in methods and reporting across microbiome studies underscores the need for standardized ROB assessment [46]. By implementing this framework, we can more accurately evaluate studies included in systematic reviews and meta-analyses, ultimately strengthening the robustness of conclusions drawn from our research [47].

**Fig. 2:**
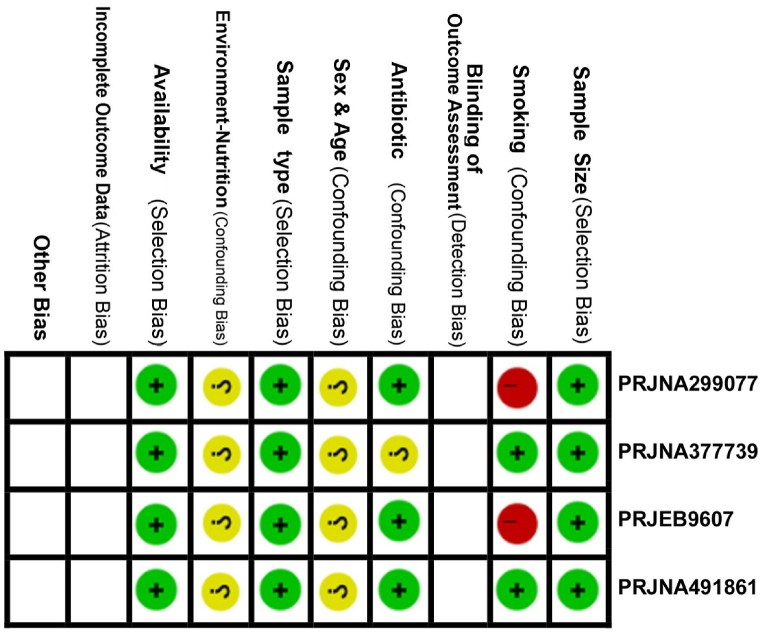
Risk Of Bias (ROB): The risk of bias (ROB) is assessed for all four datasets using three levels of judgment: low (green), moderate (yellow), and high (red). A low rating indicates a low risk of bias, a moderate rating indicates a moderate risk, and a high rating indicates a high risk of bias.

As shown in Fig. S1 and Table 1, the case and control datasets were selected based on available criteria, including disease type and status, environment, age group, V region of 16S rRNA, and other relevant factors. Additionally, each dataset will be analyzed independently for different disease states, with the results reported based on commonalities identified between the two COPD datasets. This approach ensures the highest level of similarity and minimize the risk of bias (ROB). However, areas marked in yellow and red, such as sex, nutrition, and smoking, indicate insufficient metadata regarding participants' sex, dietary habits, and smoking history (Fig. 2). This lack of data may affect the interpretation of the results, introduce potential bias, and limit the ability to fully account for these factors in the analysis. If studies with high bias show different outcomes compared to those with low bias, it could suggest that biases are influencing the findings. In this study, however, our main focus is on presenting commonalities observed in similar states of COPD.

## 3 Results

### 3.1 Data acquisition

At the start of this project, all studies on the lung microbiome using the 16S rRNA gene were screened. Studies targeted for data collection were further evaluated to confirm dataset availability. Once datasets were collected, classification was conducted on the selected data, focusing on human lung microbiome data in the V4 region from sputum samples with sufficient metadata for comparison. In Fig. S1, the flow diagram illustrates the step-by-step process of screening studies and the reasons for exclusion at each stage. It highlights the criteria for exclusion, such as animal samples, datasets outside the V4 region, absence of sputum samples, and age groups other than adults. Given the potential for bias due to variations in diseases, geographic regions, and sample sizes, steps were taken to ensure reliability. Specifically, non-COPD datasets, datasets with patients outside Britain, and datasets with small sample sizes were excluded to minimize bias in subsequent comparisons and ensure the validity of the results. The process concludes with the final selection of four datasets: two for the case group and two for the control group, which were included in the analysis. We examined the factors mentioned above in the datasets. In dataset PRJNA377739, patients were categorized based on disease status: those in a stable state, those who had experienced a single exacerbation, and those who had experienced multiple exacerbations. We conducted this classification to avoid bias and to balance the number of samples in each category. In dataset PRJNA299077, we analyzed patients in a stable state, those who had experienced an exacerbation, and those post-therapy. Since the effect of smoking was not reported in two of the datasets, we did not include it in the analyses, as we could not validate our results with those datasets.

As indicated in Table 1, the 16S rRNA genes in lung sputum were analyzed in four different datasets of COPD and healthy patients. Samples with fewer than 5000 reads were filtered out.

### 3.2 Data analysis

First, taxonomies that were not identified at the genus level were removed from the dataset. Following this, bacteria that were absent in more than 70 % of the samples were filtered out as well. Alpha and beta diversity results are shown in Figs. S3–S5. Our results show a decrease in alpha diversity from the stable state of the disease to its severe state, and an increase in alpha diversity post-therapy. Alpha rarefaction curve based on Shannon diversity and Faith_PD are shown in Figs. S5–S9.

The relative abundance of bacteria at the genus level was calculated for each sample. Similar operations were performed on all datasets individually. Among both the main and validation datasets, including healthy states, stable COPD states, and exacerbation COPD states, common bacteria with a relative frequency greater than 0.001 in more than 70 % of the samples were reported as core microbiome. Additionally, bacteria found in both the main and validation datasets with relative frequencies above and below 0.001 were shown in Table 2.

**Fig. 3:**
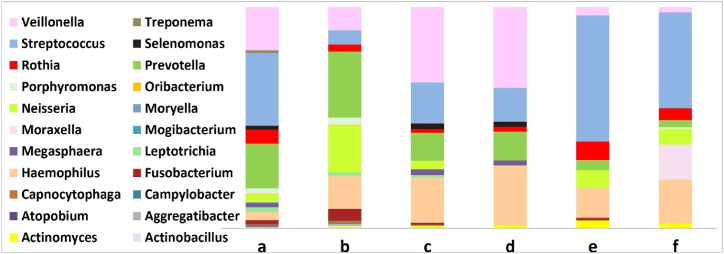
Relative abundance of the core microbiome. The relative abundance of the core microbiome at the genus level is shown in the bar chart for: a) Healthy (main dataset); b) Healthy (validation dataset); c) Stable state of COPD (main dataset); d) Exacerbation state of COPD (main dataset); e) Stable state of COPD (validation dataset); f) Exacerbation state of COPD (validation dataset).

As mentioned in Table 2 and Fig. 3, the amount of core microbiome decreases with increasing illness severity, and healthy samples have a broader range of core microbiome. The core microbiome unique to both healthy datasets include *Campylobacter*, *Leptotrichia* and *Porphyromonas*. *Fusobacterium* is observed as a core microbiome in the healthy and stable state of COPD in all mentioned datasets but is not found in the exacerbation state of COPD in both datasets. *Actinomyces*, *Prevotella*, *Streptococcus*, *Neisseria*, *Haemophilus*, *Rothia,* and *Veillonella* were identified as core respiratory microbiome members in all four datasets.

### 3.3 Correlation detection and statistical analysis

Table 3 displays the differential correlations between pairs of bacteria that exhibited an increase during the process of disease exacerbation. To rigorously control for this correlation between two bacteria across different disease states, we employed the ANOVA model. Our investigation revealed significant differences in the transitions between two bacterial pairs, *Bifidobacterium* and *Lactobacillus*, from healthy to stable and stable to exacerbation states in both the original and validation datasets (with p-values below 0.01) in Fig. 6(a and b). However, the transition from one exacerbation to multiple exacerbations in the original dataset did not show significance (p-value = 0.17). In the validation dataset, the transition from exacerbation to post-therapy demonstrated significant differences (with p-values below 0.01). Furthermore, among other pairs of bacteria across all states, no significant differences were observed.

### 3.4 Co-occurrence network

**Fig. 4:**
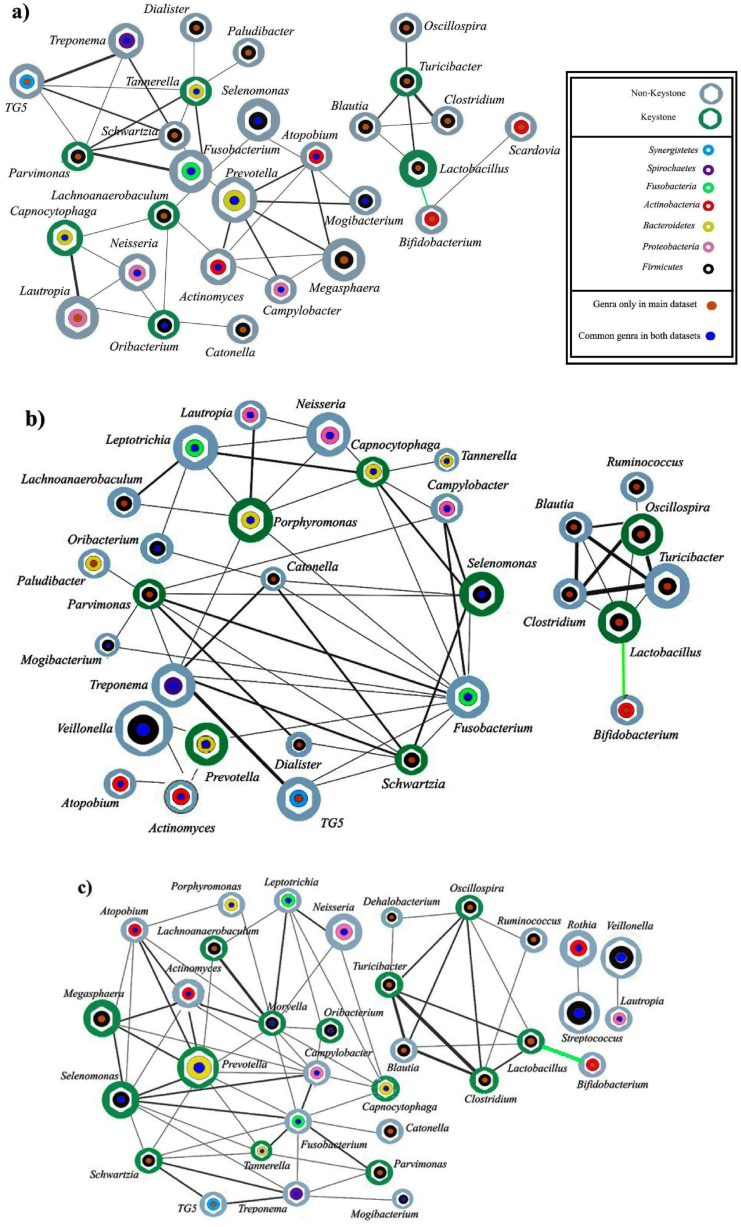
Co-occurrence network in respiratory diseases. a) Stable state of COPD (in the main dataset); b) One exacerbation of COPD (in the main dataset); c) Two or more exacerbations of COPD (in the main dataset). The nodes represent bacteria at the genus level. The size of the nodes indicates the genera's average relative abundance. Edges show a correlation above 0.6 in bacterial abundance. Green edges indicate an important correlation between genera in disease severity. The green nodes represent keystone genera. The blue circle inside the nodes represents the common bacteria in both the main and validation datasets. The phyla are represented in different colors.

Previous research on the respiratory microbiome has focused more on the diversity and abundance of bacteria in diseases than their interactions, which seem crucial in microbiome research. Fig. 4(a and b and c) depicts the co-occurrence networks constructed with pairwise correlation coefficients (>|0.6|, p-value<0.01).

The correlated bacteria in human lung sputum, according to their relative frequency at the genus level, are shown in Fig. 4(a and b and c). It was postulated that the rewiring of nearby nodes in various illness stages may cause the distinction of diseases in co-occurring networks. As the severity of the disease increases, the number of nodes, edges, and connectivity in the network also increases in both the main and validation datasets. For example, in the main dataset, the number of nodes is 28 in the stable state, 30 in the one exacerbation state, and 33 in the multiple exacerbation state. Similarly, the number of edges is 98 in stable mode, 118 in single exacerbation mode, and 144 in multiple exacerbation mode.

### 3.5 Keystone identification

Whenever the co-occurrence network is created with a correlation >|0.6|, five features are computed for each node: Degree above 2, Clustering Coefficient (CC) above or equal to 0, Closeness Centrality above 0.3, Topological Coefficient above 0.3, and Betweenness Centrality above 0. According to the above topological characteristics, and biological criteria (anaerobic bacteria are considered in this study), keystone genera were selected for each one. Finally, the keystone genera reported in Table 4 are: *Porphyromonas* in patients that have one exacerbation, and *Clostridium*, *Moryella* and *Megasphaera* in patients with more than one exacerbation were exclusive. These exclusive keystones have a higher degree in the network. *Parvimonas, Capnocytophaga* and *Lactobacillus* were common in both stable and exacerbation states of COPD. When comparing the main and validation datasets, we observed two keystone genera, *Tannerella* and *Oribacterium*, consistently present in a stable state of COPD in both datasets. Additionally, genera such as *Porphyromonas*, *Capnocytophaga*, *Selenomonas*, and *Prevotella*, along with *Tannerella* and *Oribacterium*, were found in exacerbation states of COPD in both the main and validation datasets.

**Table 4:** **A correlation network-based panel of keystone genera present in the sputum.** Five network topological characteristics, and biological criteria (anaerobic bacteria), are considered in all nodes in Fig. 4(a and b and c) to candidate the keystone genera. Keystone genera found exclusively in one network but not in the others were identified and shown in star.

Diseases	Keystone	Betweenness Centrality	Closeness Centrality	Clustering Coefficient	Degree	Topological Coefficient
**Stable COPD**	***Parvimonas***	0.06	0.42	0.7	5	0.48
	***Lachnoanaerobaculum***	0.42	0.49	0	4	0.32
	***Oribacterium***	0.18	0.37	0.16	4	0.45
	***Capnocytophaga***	0.08	0.35	0.33	3	0.6
	***Tannerella***	0.22	0.42	0.33	6	0.42
	***Lactobacillus***	0.53	0.66	0.33	3	0.53
	***Turicibacter***	0.43	0.66	0.33	4	0.5
**One Exacerbation of COPD**	*Parvimonas*	0.14	0.48	0.32	8	0.39
	*Schwartzia*	0.05	0.48	0.52	7	0.42
	*Selenomonas*	0.03	0.5	0.6	6	0.41
	*Prevotella*	0.25	0.45	0.33	3	0.38
	*Capnocytophaga*	0.17	0.51	0.33	7	0.33
	*Lactobacillus*	0.33	0.86	0.6	5	0.68
	*Oscillospira*	0.33	0.86	0.6	5	0.68
	*Porphyromonas*∗	0.20	0.51	0.33	7	0.31
**More than One Exacerbation of COPD**	***Parvimonas***	0.003	0.44	0.66	3	0.58
	***Lachnoanaerobaculum***	0.007	0.46	0.66	3	0.57
	***Oribacterium***	0.003	0.43	0.66	3	0.61
	***Selenomonas***	0.11	0.61	0.5	9	0.38
	***Lactobacillus***	0.28	0.77	0.6	5	0.66
	***Clostridium∗***	0.09	0.77	0.7	5	0.63
	***Prevotella***	0.13	0.64	0.46	10	0.36
	***Schwartzia***	0.06	0.53	0.6	6	0.44
	***Capnocytophaga***	0.045	0.54	0.6	5	0.41
	***Tannerella***	0.013	0.5	0.7	5	0.51
	***Oscillospira***	0.24	0.87	0.53	6	0.55
	***Turicibacter***	0.09	0.77	0.7	5	0.63
	***Moryalla∗***	0.23	0.64	0.31	11	0.31
	***Megasphaera∗***	0.001	0.48	0.9	5	0.56

### 3.6 Pattern candidate

**Fig. 5:**
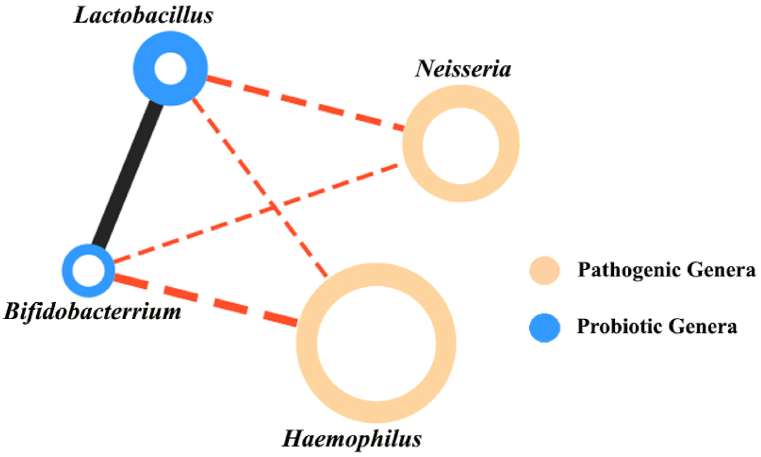
**The correlation between two probiotic genera and pathogenic genera**. The size of the nodes indicates the genera's average relative abundance Black edges show a positive correlation above 0.6, while red edges show a negative correlation above 0.4. The thickness of the edges shows the intensity of the correlation.

Upon examination of Table 3 and the results of the differential correlation between different disease states, it was discovered that the two probiotic bacteria, *Lactobacillus* and *Bifidobacterium*, exhibit a positive correlation exceeding 0.6 during COPD exacerbation. Furthermore, we investigated the negative correlations between these two probiotics and other bacteria. According to Fig. 5, it was revealed that these two bacteria have a negative correlation exceeding 0.4 with two pathogenic bacteria, *Neisseria* and *Haemophilus*.

**Fig. 6:**
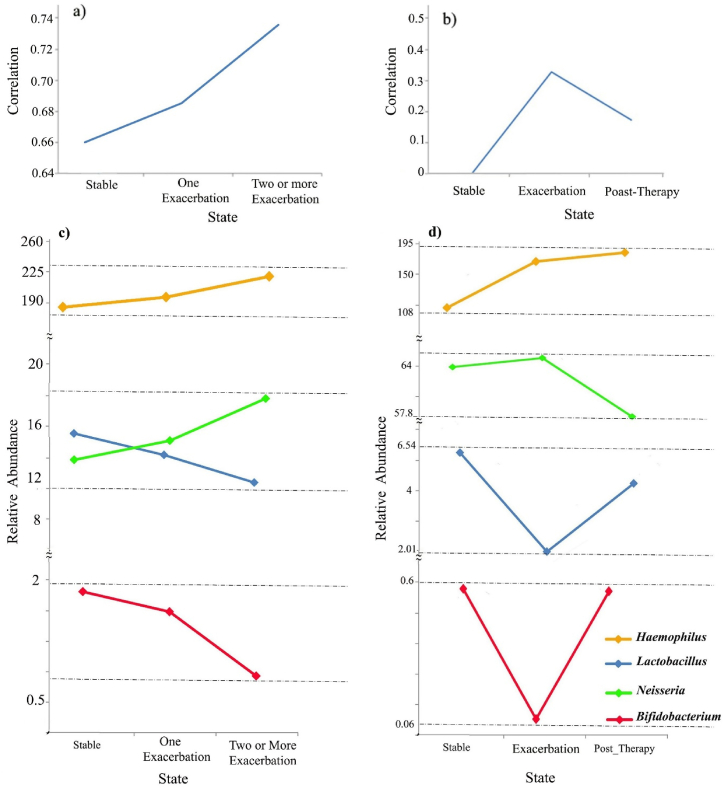
**Correlation and abundance of bacteria in the COPD exacerbation process in two main and validation datasets**. a) Correlation between *Lactobacillus* and *Bifidobacterium* in the main dataset; b) Correlation between *Lactobacillus and Bifidobacterium* in the validation dataset; c) abundance of *Lactobacillus*, *Bifidobacterium*, *Neisseria* and *Haemophilus* in the main dataset; d) abundance of *Lactobacillus*, *Bifidobacterium*, *Neisseria* and *Haemophilus* in the validation dataset.

As depicted in Fig. 6(a and b), with the escalation of disease severity, there is a notable increase in the correlation between *Lactobacillus* and *Bifidobacterium* bacteria. However, the frequency of these two bacteria decreases as the disease severity progresses, as illustrated in Fig. 6(c and d). Moreover, the negative correlation between these two probiotic bacteria and the two pathogens *Neisseria* and *Haemophilus* is shown in Fig. 5. We also indicate a significant increase in the frequency of these two pathogenic *Proteobacteria* was observed in exacerbation of COPD in both the main and validation datasets (Fig. 6(c and d)).

## 4 Discussion

In this study, we calculated the relative abundance of bacteria at the genus level in the four different cohorts of COPD and healthy individuals. We compared the lung core microbiome in stable and exacerbation states of COPD, as well as in healthy individuals. Besides, network analysis and correlation-based analysis were done to reveal microbiome interactions and keystone genera in stable and exacerbation states of COPD. We introduce a specific panel (Table 4) based on the correlation network of genera in COPD. This panel contains keystone genera for each disease state, derived from the topological and biological parameters of the microbiota. We then identified specific keystone genera for each disease state that are not found in other states. Our results showed that these keystone disease bacteria play an active role in the exacerbation of COPD. These findings provide distinct patterns for the differential diagnosis of different states of COPD in the future. We investigated all significant correlations in different health states, stable conditions and exacerbations of COPD, and compared the results with another dataset for validation. We found a significant correlation between two probiotic bacteria, *Bifidobacterium* and *Lactobacillus*, in the exacerbation of COPD. These two probiotics exhibited a negative correlation with two important pulmonary pathogenic bacteria simultaneously. This finding suggests that the concurrent use of these two probiotics could serve as potential drug candidates for the treatment or prevention of COPD exacerbations and warrants consideration in future clinical trials.

Our results show a decrease in alpha diversity from the stable state of the disease to its severe state, and an increase in alpha diversity after post-therapy (Figs. S3 and S4), consistent with previous studies that demonstrated a decline in alpha diversity from healthy to disease states [22,48]. We identified a decrease in the diversity of the lung core microbiome during exacerbation of COPD (Fig. 3). This finding agrees with other studies that reported a decline in genus diversity during disease progression [22,25,49]. As demonstrated in Table 2, a significant degree of similarity was found between healthy individuals and COPD at the genus level, including *Veillonella*, *Prevotella*, *Streptococcus,* and *Haemophilus* which is consistent with previous studies [50,51]. Our result in Fig. 3 shows the high abundance of *Prevotella* in a healthy lung microbiome, which lowers the risk of various lung illnesses. This can be explained by the ability of *Prevotella* to suppress the colonization of *Proteobacteria*, thereby promoting the immune system's response against infection [52,53]. Fig. 3 shows *Haemophilus* has high abundance levels corresponding to COPD severity. *Haemophilus* can directly induce inflammation by recruiting neutrophils and increasing mucus production. Elevated sputum CXCL8/IL-8 levels are associated with high COPD severity [54]. At the phyla level, studies have shown that the most predominant microbiome phyla in the healthy lung are *Firmicutes*, *Proteobacteria*, and *Bacteroidetes*, respectively [50,51]. By considering the phylogenetic tree (Fig. S2), we observed the same phyla in both the healthy group and the COPD group (Table 2 and Fig. 4(a and b and c)).

Our results for the co-occurrence network and topological centrality properties revealed that facultative anaerobes functioned as keystone bacteria in the lung disease microbiome, including *Firmicutes* and *Bacteroidetes* at the phylum level (Fig. 4(a and b and c)). Our results are supported by other studies, including the study of Silveira on ecological networking to predict keystone bacteria, which indicated that anaerobic mucus-degrading bacteria are keystone species in cystic fibrosis [13,14]. In our study, the predicted keystones from microbial networks partitioned the community into an anaerobic group and *Proteobacterial* pathogens (Fig. 4(a and b and c)). It is likely that this structure is driven by different carbon sources and the metabolic effects on the oxygen gradient and pH of the airway environment [14]. In a study by Pragman et al., it was shown that the presence of anaerobic members of the phyla *Firmicutes, Bacteroidetes*, and *Fusobacteriota* in the air-rich condition of the COPD lung could be explained [10]. Sugar fermentation by anaerobes lowers the pH of the environment, which promotes the growth of other fermentative bacteria that are more tolerant and successful at lower pH and creates microenvironments suitable for continued growth [14]. This may explain the positive correlation of *Firmicutes* with other anaerobic bacteria in constructed ecological networks. As the severity of the disease progresses, there is an observed increase in the number of edges and connectivity within the network in both the main and validation datasets. This finding is consistent with previous research. For instance, in the study by Shi et al., it was noted that both the connectivity and the number of connected edges significantly increased during the disease state compared to the healthy state [17]. The exclusive keystones, shown with a star in Table 4, have a higher degree in the network, indicating their greater importance compared to other keystones in the same state.

As shown in Fig. 4(a and b and c), there is a recurring sub-network of anaerobic bacteria in all these diseases, which is highlighted in green in the network corresponding to each disease. The keystone genera of the correlation network positively correlated with other anaerobic bacteria and affected their growth synergistically. The reason for the positive correlation between these two groups can be the dominants of anaerobic mucus-degrading bacteria that produce metabolites such as lactic acid, citric acid, and acetic acid during fermentation. These compounds have favorable effects on both pathogenic and aerobic bacteria, which explains why anaerobic bacteria and *Proteobacteria* are strongly correlated in our ecological networks [13]. Our results in Table 3 show that with increasing disease severity in COPD, the positive correlation between two probiotics, *Lactobacillus* and *Bifidobacterium*, increases. Conversely, there is a negative correlation between these two probiotic bacteria and aerobic *Proteobacteria*, such as *Neisseria* and *Haemophilus* (Fig. 5). In the exacerbation state of COPD, the two genera of *Proteobacteria* phylum, *Neisseria* and *Haemophilus*, thrive and spread in the pulmonary environment (Fig. 6(c and d)). These results are consistent with previous studies [55]. As shown in Fig. 6(c and d), the frequency of *Lactobacillus* and *Bifidobacterium* probiotics decreases with the severity of COPD and the increase of inflammation in the severity of the disease. *Bifidobacterium* is a commonly found commensal bacterial genus known for its beneficial effects on maintaining homeostasis and reducing inflammation. Its depletion or absence in both humans and model organisms has been linked to autoimmune responses and disruptions in immune balance. At the cellular level, *Bifidobacterium* promotes the upregulation of suppressive regulatory T cells, helps maintain the integrity of the intestinal barrier, regulates the activity of dendritic cells and macrophages, and mitigates the activation of intestinal Th2 and Th17 immune programs [56]. In research conducted by Sun et al., it was discovered that administering the probiotic *Bifidobacterium* rescued mice from a fatal inflammatory syndrome. This rescue effect was attributed, at least partially, to the impact of the probiotic treatment on regulatory CD4^+^ cells, which oversee metabolic functions and undergo immunosuppressive alterations. These regulatory CD4^+^ T cells have been recognized as a crucial mechanism in regulating autoimmunity within both mouse and human immune systems [57]. In a study conducted by Shen et al., it was demonstrated that *Lactobacillus* reuteri attenuated the inflammatory response, alleviated pulmonary edema, restored the integrity of the intestinal barrier, and restructured the gut microbiota in mice [58]. *Lactobacillus*, as a natural immunobiotic, demonstrates remarkable immunomodulatory capabilities [59,60].

We suggest that the key to understanding the pathogenesis of lung diseases may lie in deciphering the complex interactions among the host, pathogen, and resident microbiota during lung infection. Identifying keystone species based on network criteria is approximately 85 % accurate [61]. Although computational methods have shown promising results, and the findings suggest the potential of probiotics as candidates for the treatment or prevention of COPD exacerbations, further research is needed to confirm that these results are generalizable before clinical trials can proceed. At the study and outcome level, our primary limitation was that the population was predominantly composed of older adults in Britain, which restricted our ability to examine diverse environments and include other age groups. At the review level, while certain factors were reported, the metadata of some datasets lacked detailed, sample-specific information, which limited deeper analysis. For instance, insufficient data on sex, smoking history, and dietary habits hindered a comprehensive assessment of their potential impacts on study outcomes. These limitations underscore the importance of future research that addresses these gaps by prioritizing validation across diverse populations, including variations in sex, age groups, smoking history, dietary habits, and environmental contexts. By incorporating these aspects, future studies can improve the generalizability of findings and ensure broader applicability beyond the demographic constraints of the current study.

## 5 Conclusion

In this study, we proposed a specific panel for keystone detection based on a co-occurrence network in the stable and exacerbation states of COPD. We believe that these candidate panels can provide the foundation for a multi-expert AI system for early diagnosis of possible exacerbations in COPD disease. Our findings suggest that microbiome interaction is a critical factor in disease states and that rewiring of neighboring nodes at different stages of disease is as important as determining the composition and abundance of microbiome members. A significant correlation between the two probiotic bacteria, *Bifidobacterium* and *Lactobacillus* in the exacerbation of COPD and their negative correlation with the pathogenic bacteria *Neisseria* and *Haemophilus* suggests the use of these two probiotics as therapeutic candidates to prevent exacerbations in COPD in future clinical studies.

**Table:** Abbreviation:

**COPD**	**Chronic Obstructive Pulmonary Disease**
**WHO**	**World Health Organization**
**AECOPD**	**Acute Exacerbation Chronic Obstructive Pulmonary Disease**
**CF**	**Cystic Fibrosis**
**OTU**	**Operational Taxonomic Unit**
**PCoA**	**Principal Coordinate Analysis**
**CXCL8**	**C-X-C Motif Chemokine Ligand 8**
**Th2**	**T helper 2**
**Th17**	**T helper 17**
**IL-8**	**Interleukin 8**

## CRediT authorship contribution statement

**Azadeh KavianFar:** Writing – original draft, Investigation, Formal analysis, Data curation. **Hamidreza Taherkhani:** Writing – original draft, Investigation, Formal analysis, Data curation. **Hossein Lanjanian:** Writing – review & editing, Validation, Conceptualization. **Sargol Aminnezhad:** Writing – review & editing, Methodology. **Ali Ahmadi:** Writing – review & editing, Conceptualization. **Sadegh Azimzadeh:** Writing – review & editing, Validation. **Ali Masoudi-Nejad:** Writing – review & editing, Validation, Supervision, Resources, Project administration, Conceptualization.

## Consent for publication

This article does not contain any individual person data in any form.

## Ethics approval and consent to participate

This article does not involve any new studies of human or animal subjects performed by any of the authors.

## Data availability statement

The raw data used in this study are from following databases and available at

https://www.ebi.ac.uk/ena/browser/view/PRJNA377739?show=reads

https://www.ebi.ac.uk/ena/browser/view/PRJEB9607?show=reads

https://www.ebi.ac.uk/ena/browser/view/PRJNA491861?show=reads

https://www.ebi.ac.uk/ena/browser/view/PRJNA299077?show=reads.

## Funding

No funding was received for this work.

## Declaration of competing interest

The authors declare that they have no known competing financial interests or personal relationships that could have appeared to influence the work reported in this paper.
