# Supplementary material for: Keystone bacteria dynamics in chronic obstructive pulmonary disease (COPD): Towards differential diagnosis and probiotic candidates
Source: Heliyon. 2025 Feb 14;11(4):e42719. doi: 10.1016/j.heliyon.2025.e42719 (PMC11876909; doi:10.1016/j.heliyon.2025.e42719)
Supplement: Multimedia component 1 [file mmc1.docx]

**Supplementary file 1:** The details of " *Keystone Bacteria Dynamics in Chronic Obstructive Pulmonary Disease (COPD): Towards Differential Diagnosis and Probiotic Candidates*”

**
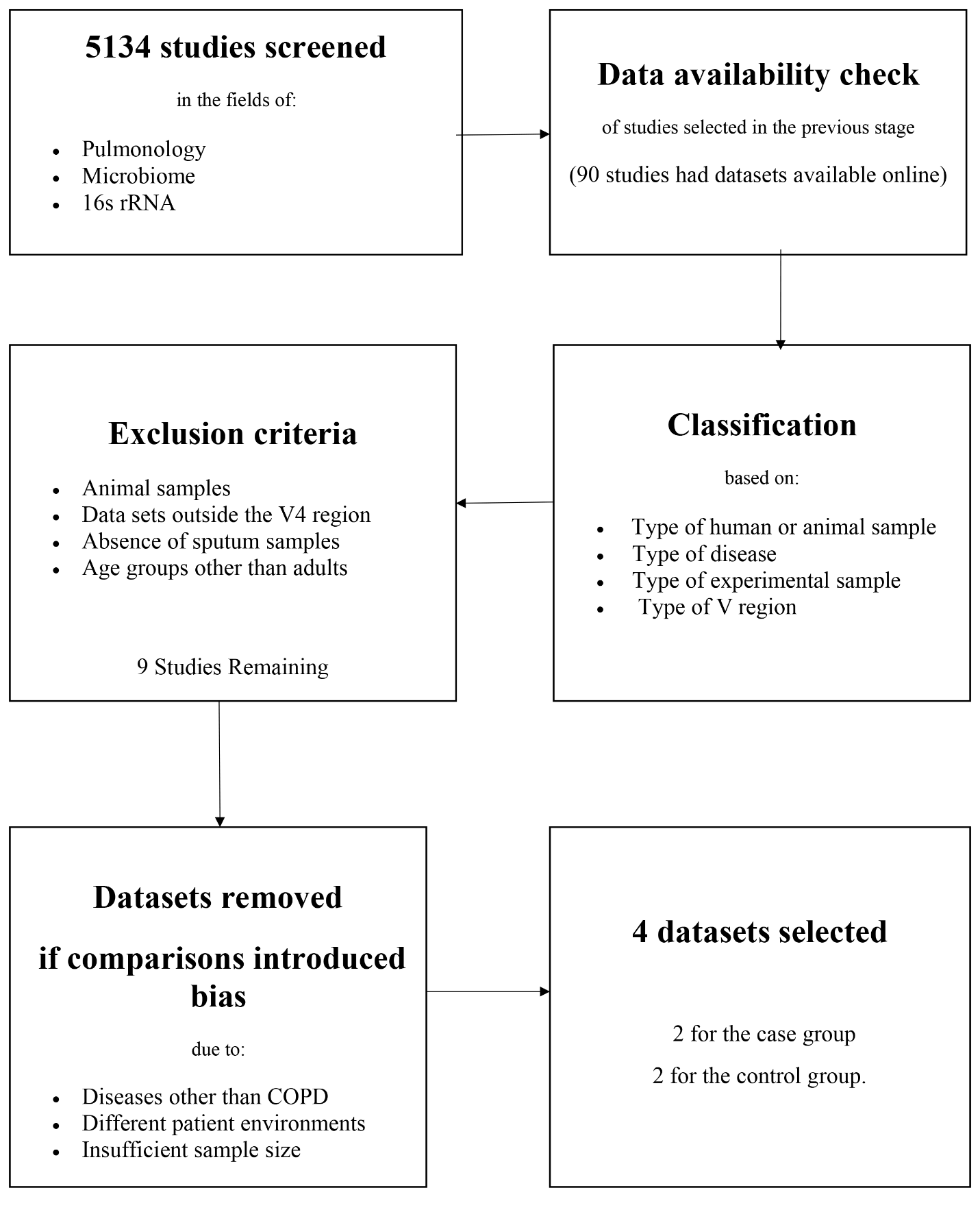
**

**Figure S1:** **Flow Diagram of the Study Screening Process;** this flow diagram illustrates the step-by-step process of screening studies and the reasons for exclusion at each stage. It shows the progression from the initial identification of studies through various screening phases, specifying the exclusion criteria at each step. The process concludes with the final selection of studies included in the analysis.


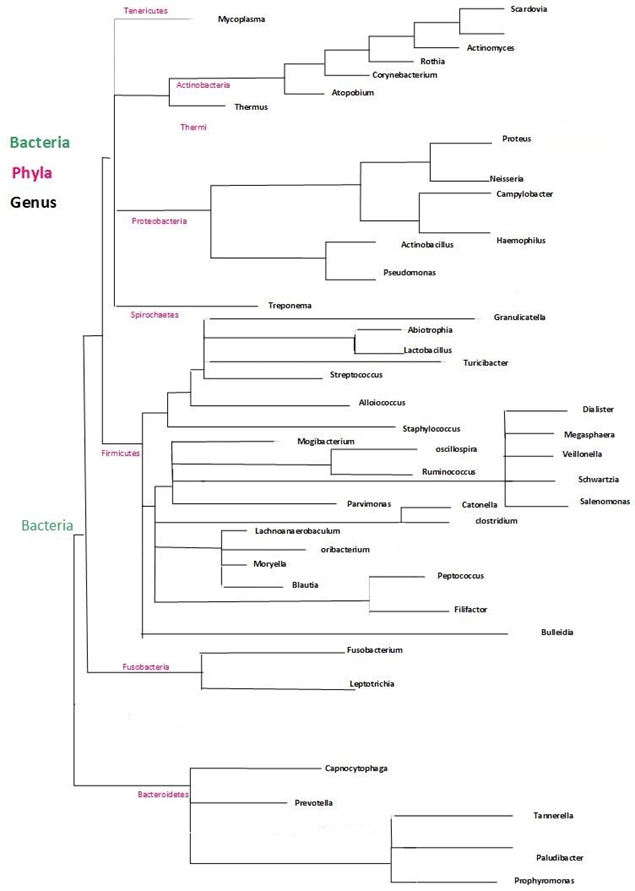


**Figure S2**: **Phylogenetic tree;** the evolution of a species, creature, or gene is illustrated by an evolutionary tree, called phylogeny. Species, creatures, or genes all belong to this branch of evolution. The tips (OTUs) in the tree are annotated with dots. Each tip is annotated with a dot for each sample where the OTU was observed. To analyze and display phylogenetic sequencing data for previously clustered OTUs, use Phyloseq, which takes phylogenetic sequencing data and stores it. Data visualization can be done with ggplot2.


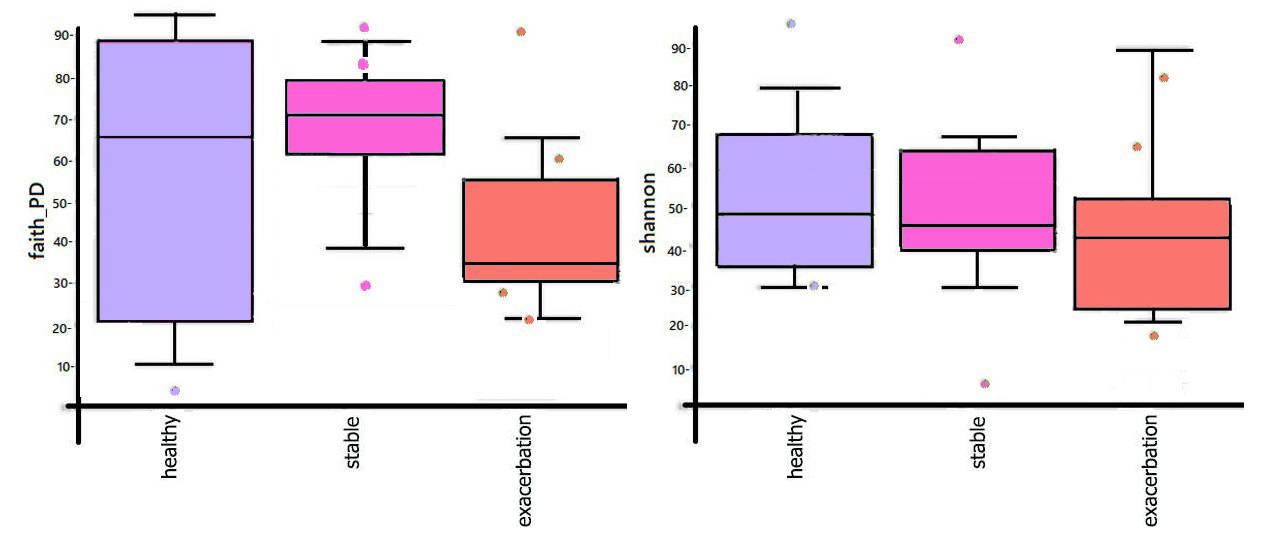


**Figure S3**: Alpha Diversity in main datasets.


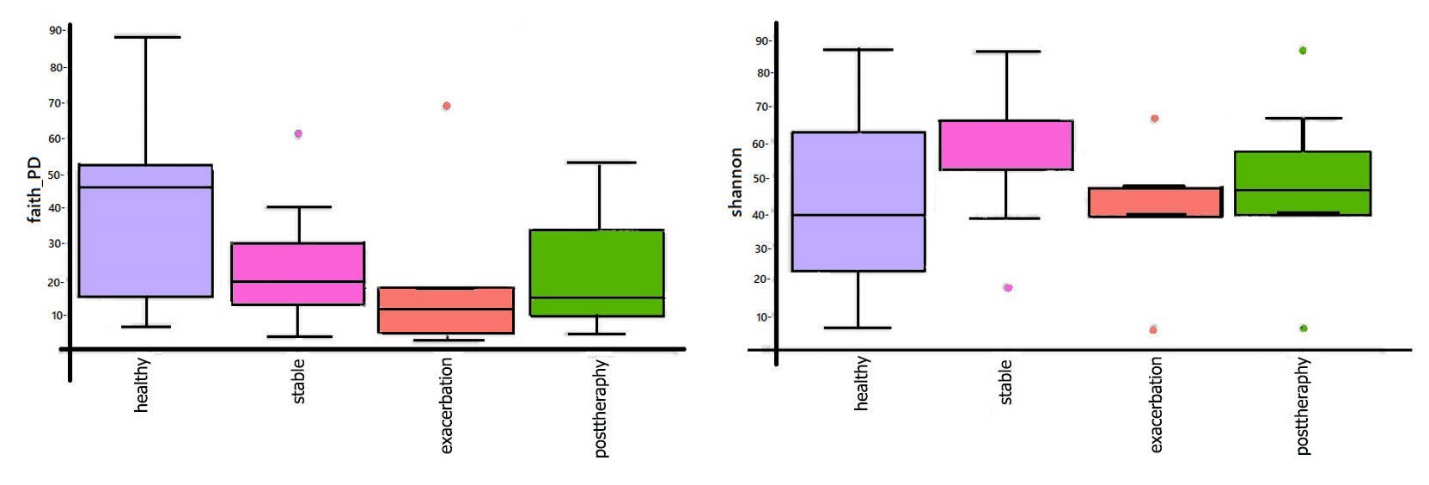


**Figure S4**: Alpha Diversity in validation datasets.


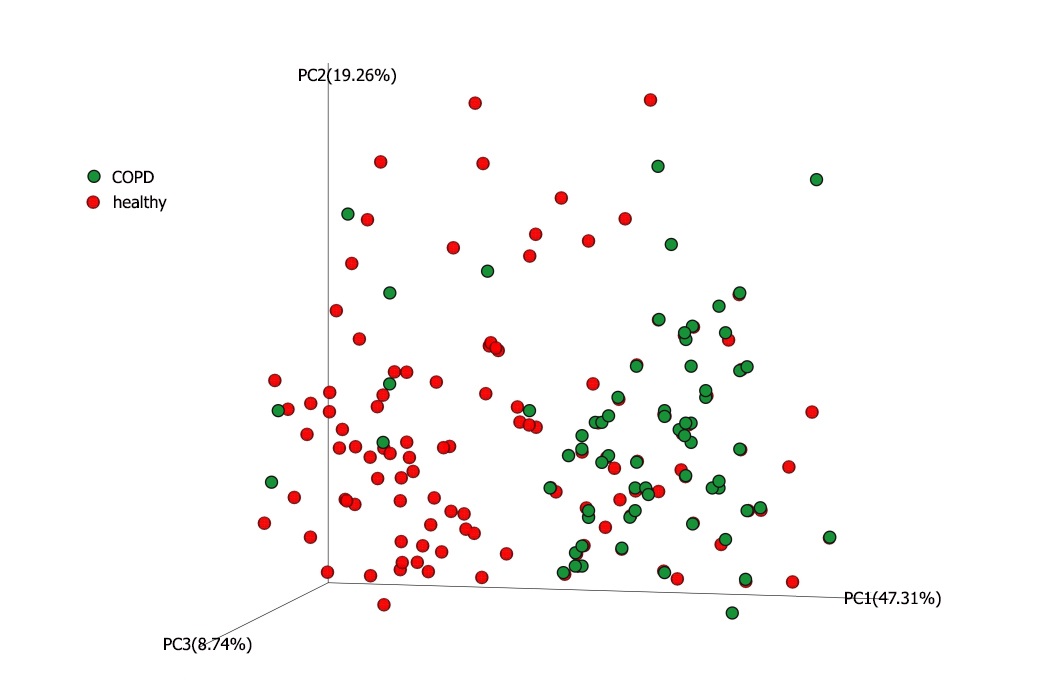


**Figure S5**: Beta Diversity based on principal coordinate analysis (PCoA) plots in main datasets: The red nodes represent healthy individuals, while the green nodes represent COPD patients.


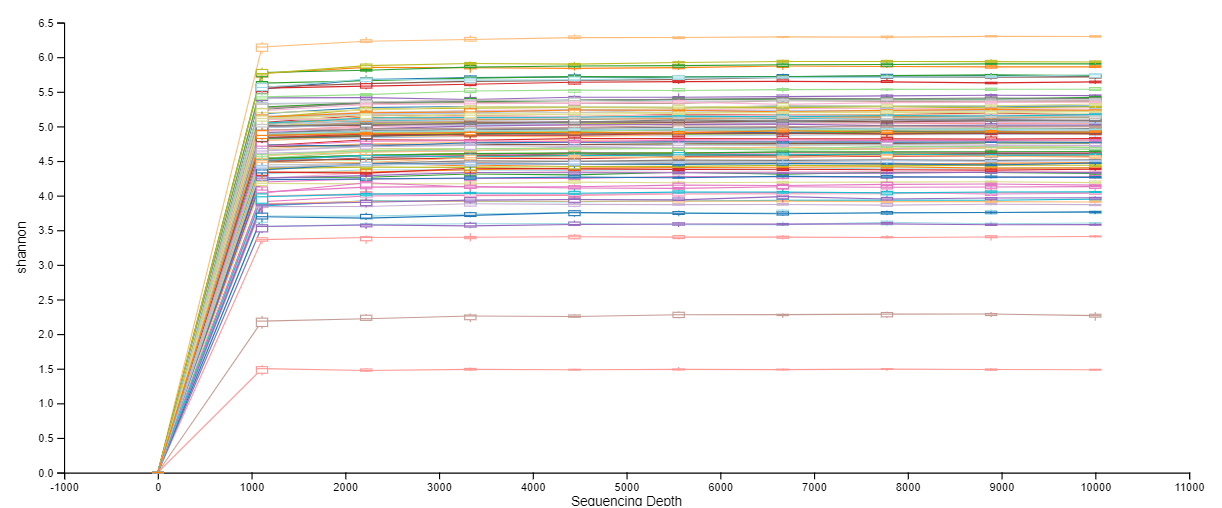


**Figure S6**: Alpha Rarefaction curve of healthy (main dataset) based on Shannon Diversity.


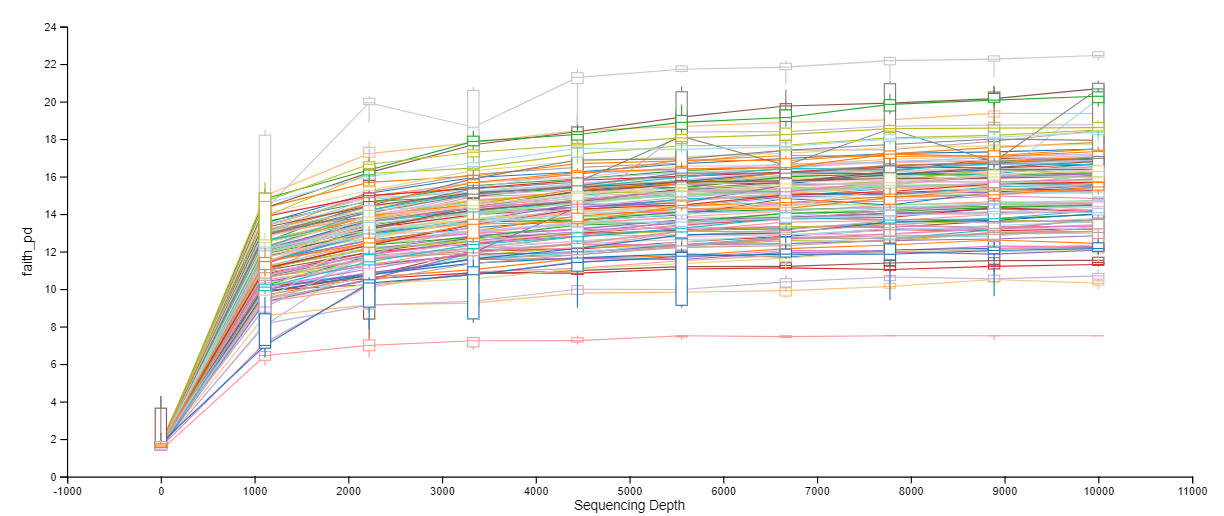


**Figure S7**: Alpha Rarefaction curve of healthy (main dataset) based on Faith_PD.


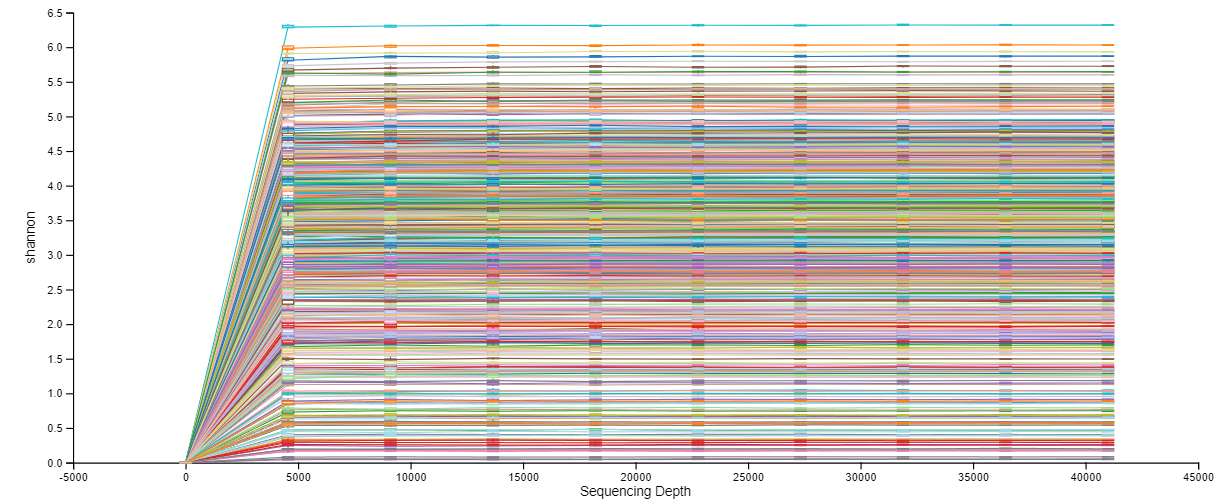


**Figure S8**: Alpha Rarefaction curve of COPD (main dataset) based on Shannon Diversity.


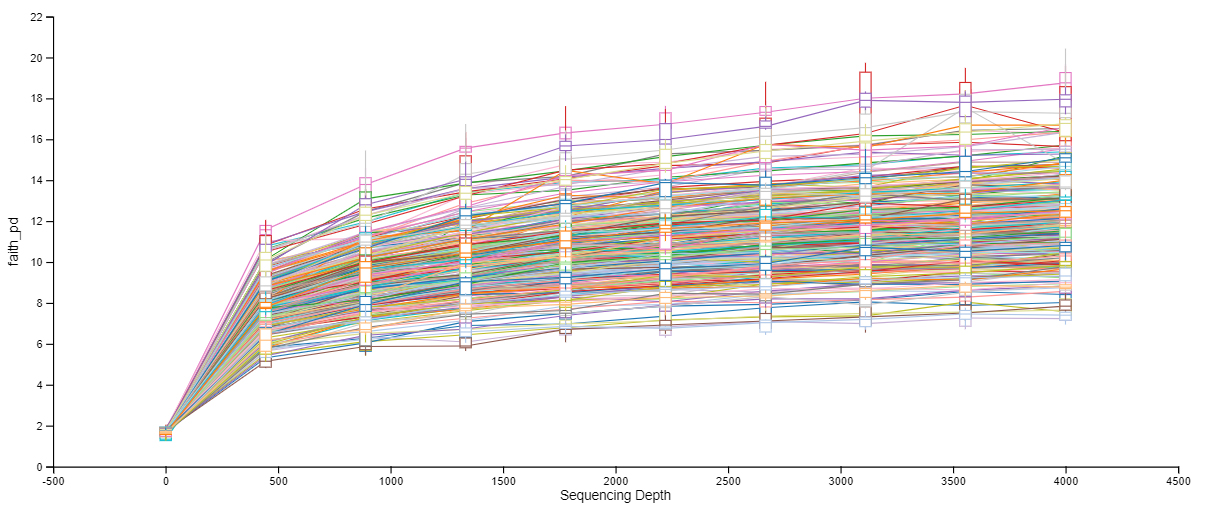


**Figure S9**: Alpha Rarefaction curve of COPD (main dataset) based on Faith_PD
